# A case report of primary breast angiosarcoma: An uncommon malignancy

**DOI:** 10.1016/j.radcr.2025.05.043

**Published:** 2025-06-12

**Authors:** Ayesha Afzal, Muhammad Junaid Tahir, Asma Asghar, Hira Yaqub, Anis Ur Rehman, Aamer Iftikhar

**Affiliations:** a Department of Radiology, Shaukat Khanum Memorial Cancer Hospital and Research Center, Lahore, Pakistan; b Department of Pathology, Shaukat Khanum Memorial Cancer Hospital and Research Center, Lahore, Pakistan

**Keywords:** Primary angiosarcoma, Ultrasound, Vascular, Tumor markers, Atypical endothelial cells

## Abstract

Angiosarcoma of the breast is a rare and aggressive form of cancer that originates from vascular or lymphatic tissues, representing only 0.04% of malignant breast lesions. Variance in the clinical, pathological, and radiological presentations often leads to challenges in diagnosis. We present a case of a 32-year-old lactating female having a painless swelling in her right breast for 6 months, with a significant increase in size in 1 month. Clinical assessment indicated the presence of a mass at the superior edge of the areola, with associated bluish skin discoloration. Ultrasonography revealed a vascular lesion characterized by mixed echogenicity. A core biopsy confirmed the diagnosis of angiosarcoma. Imaging studies, including computed tomography (CT) and bone scans, ruled out distant metastases. Subsequently, surgical excision was performed, and histopathological analysis reaffirmed the diagnosis of angiosarcoma. Radiotherapy was deemed unsuitable due to the potential risk of recurrence or radiation-induced secondary angiosarcoma. This case highlights the necessity of a multidisciplinary approach in optimizing diagnosis and treatment, with surgical excision playing a pivotal role in disease management.

## Introduction

Angiosarcoma is an aggressive malignant tumor originating from lymphatic or vascular tissues accounting for 2% of all soft tissue sarcomas in adult humans. Angiosarcoma is the most common sarcoma of all breast cancers and is a rare malignancy [1]. Primary angiosarcoma is extremely rare as 0.04% of all malignant breast lesions account for 20% of all angiosarcomas of the breast with an incidence rate of 17 per million women. It is presented mostly in the third or fourth decade of life and is usually misdiagnosed attributing to the nonspecific and varied clinical, pathological, and radiological presentation [2]. We presented a case of primary angiosarcoma of the breast in a young adult female along with the literature available on presentation, diagnosis, and therapeutic modalities.

## Case presentation

A 32-year-old lactating female presented with the complaint of swelling in the right breast for the last 6 months with a gradual increase in the size of the swelling for 1 month, having no association with pain or bleeding. She had no history of any co-morbidities or breast cancer in the family. On examination, there was a 4 × 4 cm swelling in the superior areolar margin of the right breast with overlying bluish skin discoloration, and the clinical score of suspicion was P4. The examination of the left breast and bilateral axillae was unremarkable.

**Fig. 1:**
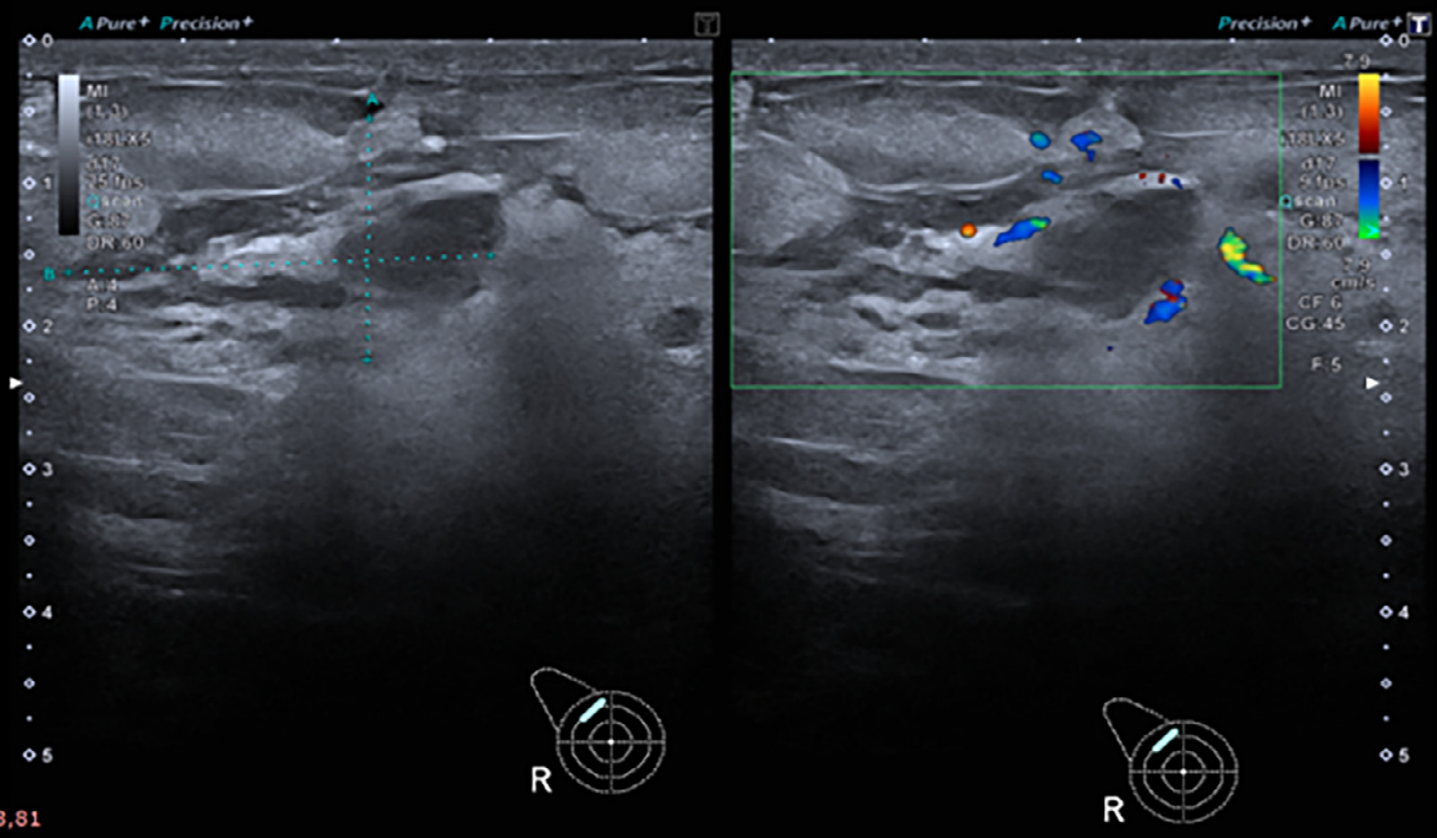
Ultrasound images showing ill-defined areas of abnormal, mixed hyperechogenic and hypoechogenic regions without a discrete mass in upper outer quadrant. Color Doppler shows hypervascularity.

On ultrasonography of the breast, an ill-defined area of mixed echogenicity in the right breast at 10-11 o'clock position measuring up to 2.7 × 1.7 cm, demonstrating vascularity on color Doppler imaging and was categorized as BIRADS 4 (Fig. 1). The left breast and bilateral axillae remained clear.

**Fig. 2:**
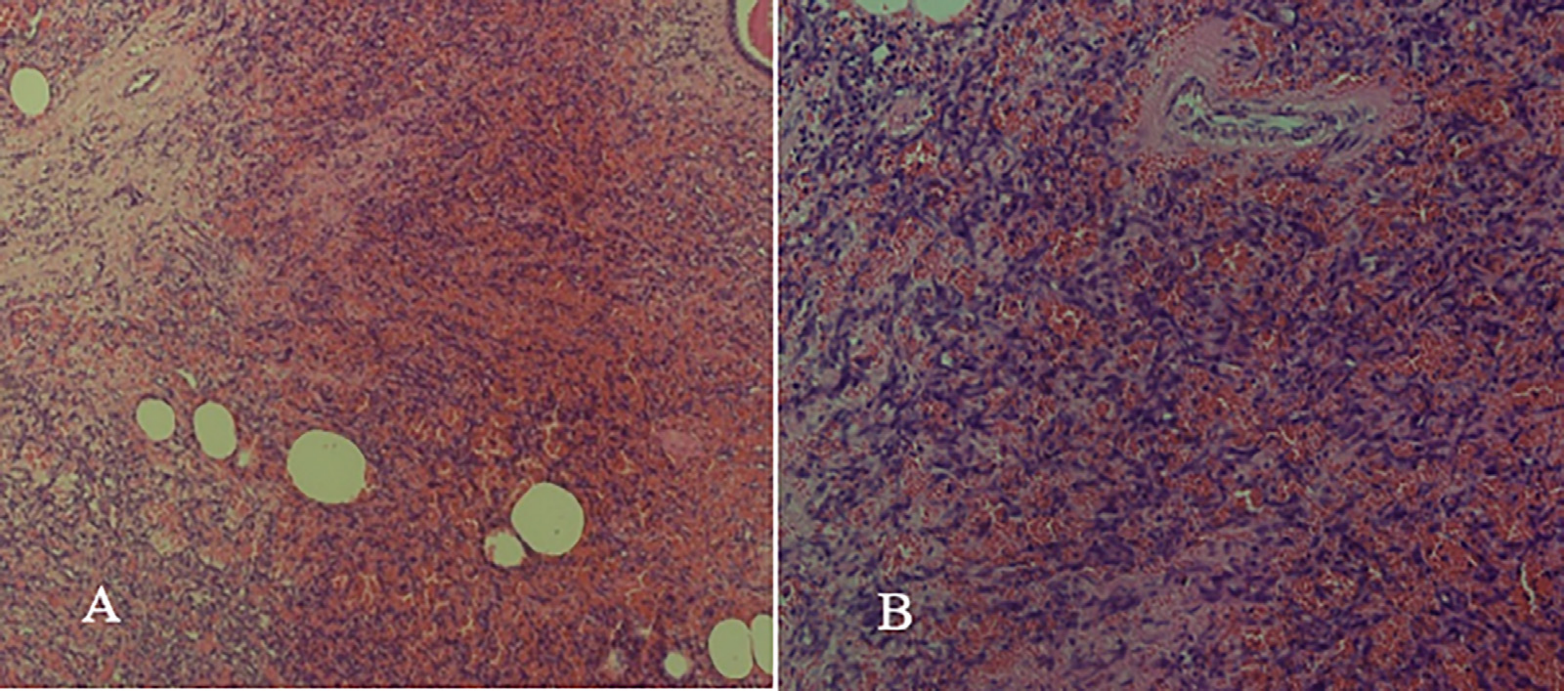
(A) Angiosarcoma resection showing well-formed vascular channels with mildly atypical spindle cells and hemorrhage in the background (HE stains: 10X). (B) Angiosarcoma with mildly atypical spindled cells (HE stains: 40X).

**Fig. 3:**
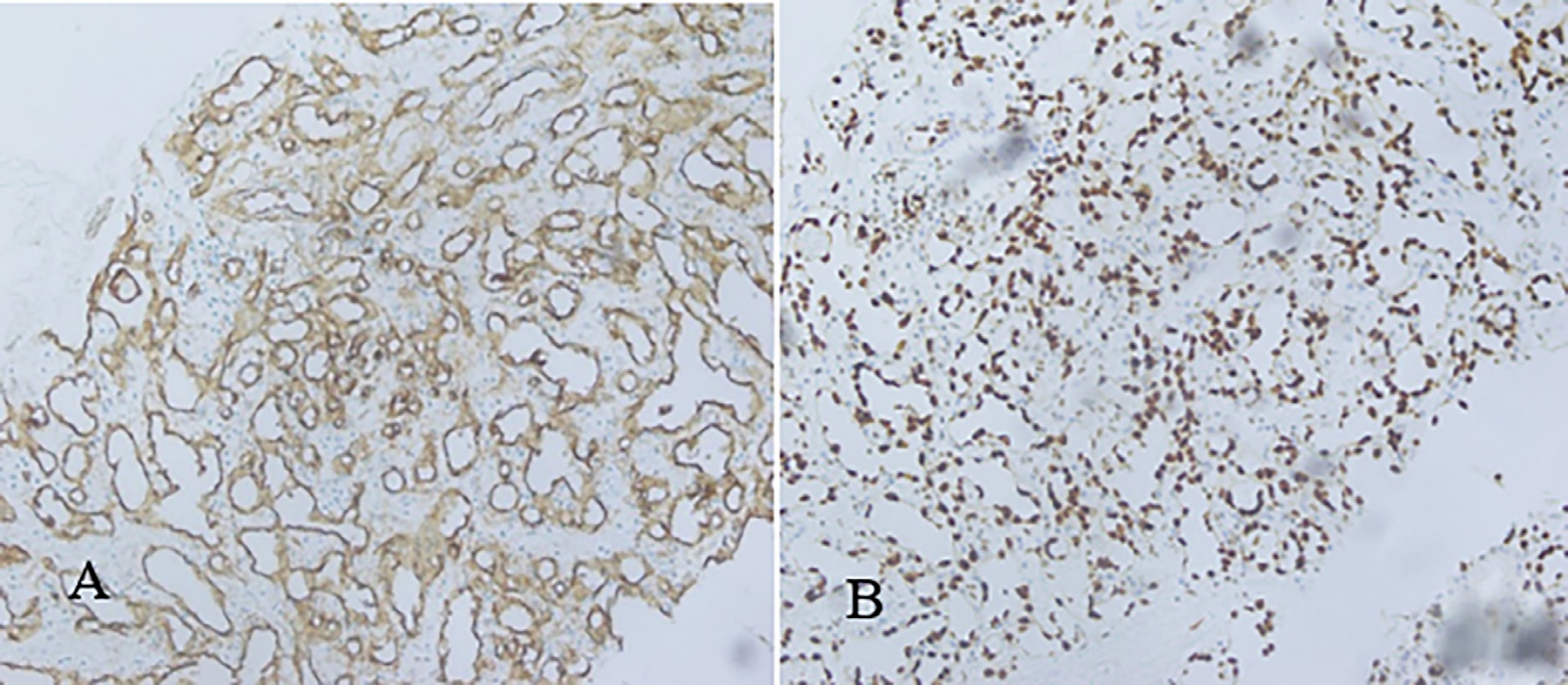
Immunohistochemical stains showed positivity to anti-CD34 antibodies and ERG that highlighted the endothelial cells. (A) Initial core biopsy showing positive staining of CD34 (40X). (B) Core biopsy showing positive nuclear staining for ERG (40X).

Ultrasound-guided core biopsy was performed for a definite diagnosis, as imaging was equivocal. The biopsy specimen with Hematoxylin & Eosin (HE) staining showed well-formed and irregular vascular channels lined by mildly atypical endothelial cells with the background of stroma comprising dense fibrous tissue. Cellular atypia was characterized by nuclear hyperchromasia, mildly increased mitotic activity, and pleomorphism. Background hemorrhage was also appreciated (Fig. 2). Immunohistochemical stains showed anti-CD34 antibody-positive around well-defined and interconnected vascular channels and ERG-positive in atypical spindle cells (Fig. 3). Features were consistent with angiosarcoma.

**Fig. 4:**
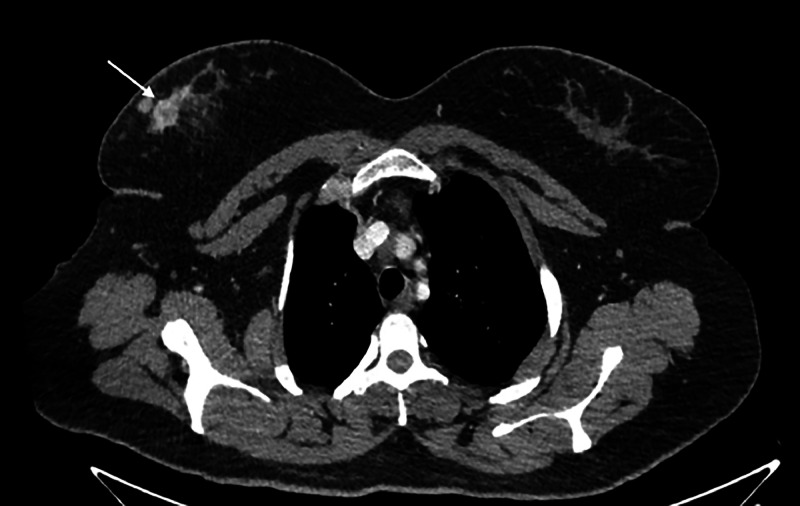
CT scan showing enhancing nodular mass (white arrow) in the upper outer quadrant of right breast with adjacent parenchymal distortion and subtle skin thickening. No distant metastasis.

After confirmation of malignancy, computed tomography (CT) chest, abdomen, and pelvis was performed as a metastatic workup before surgical resection. On CT scan, a 2.9 × 1.8 cm mass was seen in the upper outer quadrant of the right breast with distortion of surrounding parenchymal tissue and thickening of overlying skin (Fig. 4). No distant metastasis was identified on the CT scan. Furthermore, the patient's bone scan was also unremarkable. Sentinel lymph node scintigraphy showed a sentinel lymph node and a second-tier node towards the right axillary region. Magnetic resonance imaging (MRI) breast could not be performed due to limited resources.

Subsequently, right breast superior segmentectomy with sentinel lymph node dissection and Batwing mammoplasty was performed. Specimen histopathology also confirmed angiosarcoma of the breast.

**Fig. 5:**
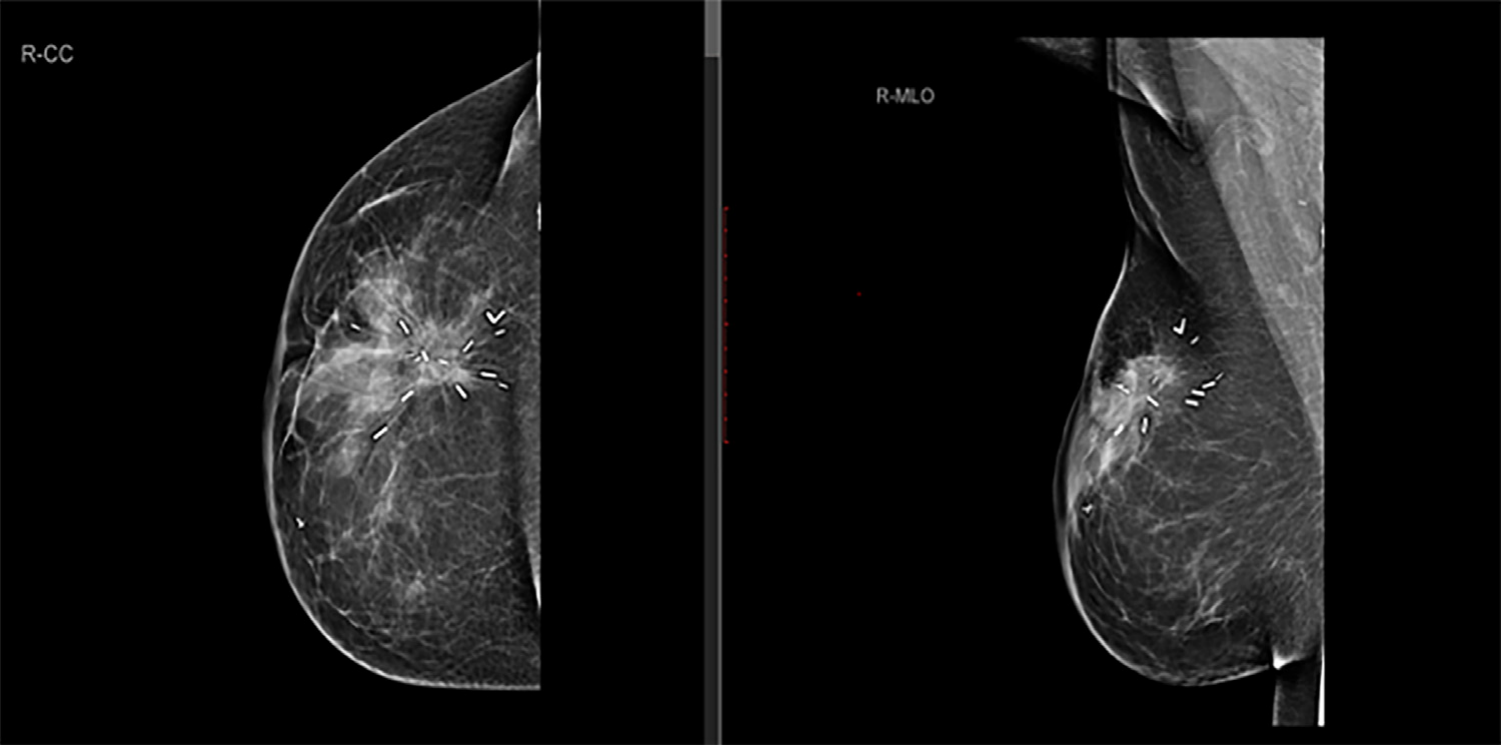
First postoperative mammogram (after 6 months of surgery) performed at showed postsurgical architectural distortion in right breast upper outer quadrant with surgical clips in place.

**Fig. 6:**
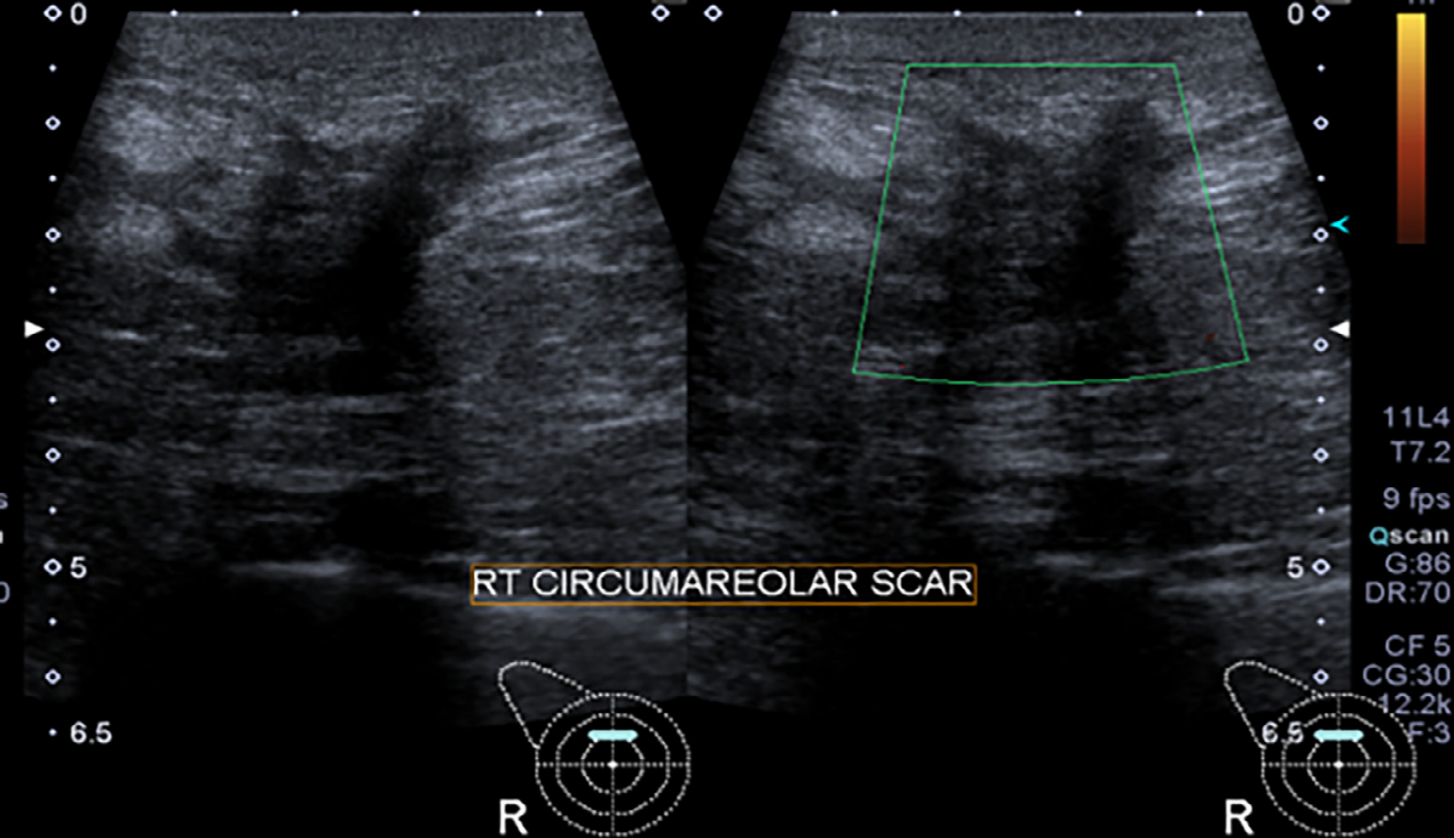
First postoperative ultrasound (performed adjunct to the mammogram) shows a mildly thickened circum-areolar scar with no suspicious nodularity or abnormal vascularity on color Doppler. Biopsy of the scar shows no evidence of recurrence.

The patient was referred to a radiation oncologist and radiotherapy treatment was refused to this patient to prevent the disease recurrence or secondary angiosarcoma formation as secondary angiosarcoma is a radiation-induced malignancy. Therefore, it was planned to keep the patient under regular surveillance for postoperative complications and recurrence. Post operative mammogram and ultrasound were performed after 6 months of surgery and both showed postsurgical changes with slightly thickened scar site; however, there was no scar site nodularity or suspicious mass (Fig. 5, Fig. 6). Primary surgeon recommended ultrasound guided core biopsy of thickened scar to rule out recurrence, and histopathology revealed only scar tissue with no evidence of sinister pathology.

## Discussion

Malignant neoplasms of the breast arising from stromal cells account for < 1% of breast malignancy. The most common sarcoma of the breast is angiosarcoma which is still a rare malignancy with an 8% incidence rate of breast sarcomas and the origin is from endovascular tissue [2]. It occurs in primary form without a known precursor, or secondary form associated with a history of irradiated breast tissue. Both forms have a malignant behavior and a poor prognosis [1].

Primary angiosarcomas are common in females with an age range from 20 to 50 years and are rarely present in males. The right breast is more commonly involved than the left [3]. In this case, we reported a young adult female of 32 years of age involving the right breast and was also currently lactating. Approximately 12% of primary angiosarcoma are diagnosed during pregnancy or the postpartum period indicating the effect of hormones, however, the majority of cases are negative for estrogen and progesterone receptors. The other potential factors responsible for rapid growth in pregnancy and lactation could be immunosuppression and placental growth factors [3,4].

Primary angiosarcoma is usually presented as a rapidly growing mass. Patients complain of a palpable lump, swelling, or fullness in the breast in contrast to the secondary angiosarcoma with presentation as a painless multifocal rash [5]. The size of the tumor ranges from 1 to 25 cm with an average size of 5.9 cm [3,6]. In this case, the mass has a size of 4 × 4 cm with the increase in size in 1 month. One-third of patients had the overlying bluish discoloration as in this patient which is attributed to the vascular nature of the tumor and involvement of the skin. Large masses of angiosarcoma could lead to platelet sequestration with hemorrhagic presentation as thrombocytopenia and consumption coagulopathy [5].

Primary angiosarcomas are mostly misdiagnosed on mammography due to nonspecific findings with usually an ill-defined, noncalcified mass along with focal asymmetry [6]. The sonographic evidence of angiosarcoma is characterized by hyperechogenicity or mixed hyper-hypoechogenicity and is attributed to the vascular nature of the lesion having multiple interfaces of the blood vessels [7]. Ultrasound findings of this case was also a lesion with mixed echogenicity and vascularity on Doppler imaging. The presence of anechoic features resulting from hemorrhage and necrosis is also an ultrasound feature of angiosarcoma [8].

CT has a very limited role in the diagnosis of breast angiosarcoma and is used for staging purposes to assess distant metastasis. Lung parenchyma is the most common site for metastasis. No distant metastasis was reported on the CT scan in this case and only localized breast tissue distortion and skin thickening were found [7]. Breast angiosarcoma had specific characteristics on MRI. Angiosarcoma demonstrates elevated signal intensity on T2-weighted imaging, with notable enhancement occurring during the initial stages, and the enhancement curve typically follows a washout pattern. Large masses of angiosarcoma usually had high intensity on both T1 and T2-weighted images resulting from thrombocytopenia and hemorrhages. Hemosiderin ring could also be observed in lesions with long-term hemorrhages [9,10].

Fine needle aspiration (FNA) or core needle biopsy for histopathological diagnosis of angiosarcoma is difficult and has a false negative rate of 37% [11]. Excessive bleeding after biopsy is also evidence of vascular lesion. Morphologically, a diverse array of growth patterns and nuclear atypia can be observed. Well-differentiated angiosarcomas are characterized by interconnecting vascular channels that infiltrate adipose tissue and lobular stroma. Other architectural forms include vasoformative growth, solid growth, papillary endothelial growth, and a capillary-type structure [3]. High-grade angiosarcomas that are poorly differentiated can feature solid zones filled with numerous large neoplastic cells arranged in sheets, lacking the typical blood channels. Frequent mitotic figures and significant necrosis further complicate the diagnostic evaluation [10]. Immunohistochemistry plays a crucial role in distinguishing between angiosarcomas and invasive carcinomas. Angiosarcoma is characterized by endothelial markers such as CD 34, CD 31, ERG and factor VIII. Additionally, a higher Ki67 index is associated with poor prognosis [7]. The histological findings of this case showed well-formed and irregular vascular channels lined by anti-CD34 antibody-positive atypical endothelial channels with a background of dense fibrous tissue.

The differential diagnosis of angiosarcoma of the breast depends on the grade of the tumor. The differential diagnosis for low-grade lesions is angiomatosis, angiolipoma, hemangioma, and benign proliferative lesions. The differential diagnosis for high-grade primary angiosarcoma is both vascular and nonvascular neoplasms. Mastitis, fibromatosis, squamous cell carcinoma with sarcomatoid features, phyllodes tumor, and invasive mammillary carcinoma [7,11,12].

Surgical resection of the mass is the mainstay of treatment for primary breast angiosarcoma. Mastectomy is the preferred treatment, and breast conservation surgery could also be recommended. Axillary clearance is not advised for angiosarcoma due to the hematogenous dissemination of the tumor [13]. High-grade angiosarcoma has shown more favorable outcomes with chemotherapy. For local recurrence, the application of radiation therapy may be appropriate. However, there remains a lack of consensus regarding the use of preoperative radiotherapy in metastatic cases of angiosarcoma [3].

The prognosis of primary angiosarcoma of the breast depends on the margin involvement after surgical resection, tumor grade, and size. The 5-year disease-free survival for Grade I and Grade III primary angiosarcoma post-treatment is 76% and 15%, respectively. The common metastatic sites are the lungs, liver, and bones. Metastasis to the contralateral breast has also been reported [6,7].

## Conclusion

Primary angiosarcoma of the breast is a rare malignancy and this rare case presentation recollects the features of the tumor developing in a patient without radiation exposure. The prognosis is particularly poor and requires early diagnosis and treatment. It is rarely identified before surgery and is challenging to diagnose clinically, radiologically, or histologically. The size and location of the tumor determine the appropriate surgical intervention. The role of chemotherapy and radiation therapy for primary angiosarcoma is still unclear.

## Author contributions

A.A (Ayesha Afzal) and M.J.T conceived and designed the case report, A.A (Ayesha Afzal), H.Y, and A.A(Asma Asghar) were responsible for data collection and acquisition of data. A.A (Ayesha Afzal) and M.J.T performed the literature review and wrote the manuscript. A.U.R, A.I, and M.J.T reviewed and critically revised the manuscript. All authors have approved the final manuscript.

## Consent for publication

This case report was conducted with the approval of the institutional review board (EX-04-06-24-01) and written informed consent for the publication of this case report was obtained from the patient. All patient data were de-identified to maintain confidentiality.

## Patient consent

The consent was acquired from the patient.
